# sEMG-based automatic characterization of swallowed materials

**DOI:** 10.1186/s12938-024-01241-z

**Published:** 2024-05-17

**Authors:** Eman A. Hassan, Yassin Khalifa, Ahmed A. Morsy

**Affiliations:** 1 Biomedical Engineering Dept., Cairo University, Giza, Egypt; 2 Center for Research Computing, University of Pittsburgh, Pittsburgh, PA USA; 3 Information Technology Analytics, University of Pittsburgh, Pittsburgh, PA USA

**Keywords:** Swallowing, Bolus volume, Weight management, IMU, sEMG, Classification

## Abstract

Monitoring of ingestive activities is critically important for managing the health and wellness of individuals with various health conditions, including the elderly, diabetics, and individuals seeking better weight control. Monitoring swallowing events can be an ideal surrogate for developing streamlined methods for effective monitoring and quantification of eating or drinking events. Swallowing is an essential process for maintaining life. This seemingly simple process is the result of coordinated actions of several muscles and nerves in a complex fashion. In this study, we introduce automated methods for the detection and quantification of various eating and drinking activities. Wireless surface electromyography (sEMG) was used to detect chewing and swallowing from sEMG signals obtained from the sternocleidomastoid muscle, in addition to signals obtained from a wrist-mounted IMU sensor. A total of 4675 swallows were collected from 55 participants in the study. Multiple methods were employed to estimate bolus volumes in the case of fluid intake, including regression and classification models. Among the tested models, neural networks-based regression achieved an *R*^2^ of 0.88 and a root mean squared error of 0.2 (minimum bolus volume was 10 ml). Convolutional neural networks-based classification (when considering each bolus volume as a separate class) achieved an accuracy of over 99% using random cross-validation and around 66% using cross-subject validation. Multiple classification methods were also used for solid bolus type detection, including SVM and decision trees (DT), which achieved an accuracy above 99% with random validation and above 94% in cross-subject validation. Finally, regression models with both random and cross-subject validation were used for estimating the solid bolus volume with an *R*^2^ value that approached 1 and root mean squared error values as low as 0.00037 (minimum solid bolus weight was 3 gm). These reported results lay the foundation for a cost-effective and non-invasive method for monitoring swallowing activities which can be extremely beneficial in managing various chronic health conditions, such as diabetes and obesity.

## Background

Swallowing is an essential function in which food and liquids are transferred from the oral cavity to the stomach to provide necessary nutrients vital for human survival. Therefore, monitoring swallowing activities can be considered an ideal surrogate for tracking the ingestive behavior of an individual for purposes of observation, measurement, and control. Swallowing is the result of a well-coordinated, yet complex set of neuromuscular activities that involves more than 30 nerves and muscles [1, 2]. The swallowing process is commonly divided into three phases: oral preparatory phase, pharyngeal phase and esophageal phase. The timing and duration of each of the three phases vary according to multiple factors that include material consistency, bolus volume, age and health condition of the subject [3, 4]. However, of the three phases, the pharyngeal phase is considered the most important as many of the swallowing physiological events such as airway protection and upper esophageal sphincter opening occur in this phase [5, 6].

Automatic tracking of swallowing activities is of special importance to identify and intervene in certain health situations that include eating patterns of the elderly, food consumption disorders in type 1 diabetes, and obesity. According to the United Nations, the world population included more than 700 million persons of age 65 or older in 2019 [7]. As people grow older, they become more vulnerable to a wide variety of diseases such as cognitive, cardiac and metabolic diseases. Studies indicate that a significant number of such diseases are linked to nutrition deficiencies, which include changes in the eating and drinking patterns [8]. Such pattern changes usually have a direct influence on people’s health, especially the elderly. Diabetes is the third most prevalent chronic childhood diseases and is considered a leading cause of retinopathy, nephropathy and cardiovascular diseases later in life [9]. Also, about 40% of the diabetic population are of age 65 or older [9]. Obesity is a complex disorder that involves abnormal body weight for the corresponding height due to excessive amounts of body fat resulting, mostly, from excess calorific intake. Obesity is one of the leading causes of a set of chronic diseases, such as cardiovascular diseases, diabetes, and hypertension [10]. Common management protocols for diabetes and obesity include weight loss through management of calorie intake, being more physically active, and making other changes to routine eating habits. Management of food intake relies mainly on monitoring the acts of swallowing to characterize the amount of food/drink consumed by the subject [3, 11]. While multiple methods have been explored for the automatic characterization of various physical activities, less has been done on developing accurate and inexpensive ways for day-to-day monitoring of ingestive activities [12, 13].

Known methods for the evaluation of the physiology of swallowing include X-ray videofluoroscopy, electromyography (EMG), and more recently cervical auscultation. Videofluoroscopy is a radiographic method that is not available outside custom clinical settings and exposes subjects to radiation [4, 6, 13]. Recent research explored the monitoring of food/drink intake activity through utilizing a variety of sensing modalities associated with sensing specific movements of the head and neck region as well as the hands. For instance, swallowing sounds collected from a throat microphone and conduction microphone placed on the mastoid bone along with a strain sensor have been used for monitoring chewing and swallowing activities and determining the bolus nature being composed of solids or liquids [13, 14]. Other studies have also utilized sensors like accelerometers and strain sensors for monitoring swallowing activities [6, 15–17]. Further, with a camera that can be triggered by a set of sensors when food intake is detected, the accuracy of monitoring could be improved by up to 82.7% [18]. Another study used a proximity sensor, IMU and throat microphone to differentiate between daily activities and food intake with accuracy of 97% [19]. Wrist motion tracking was utilized as well to detect periods of eating and drinking to improve accuracy [20, 21].

Monitoring fluid intake via the use of a throat microphone or mechanical sensors was reported but without estimating the fluid intake volume [22]. Another study reported the use of a throat microphone to estimate the fluid intake volume with a reported accuracy of 80% for volumes between 5 and 15 ml per subject [23]. Swallowing and chewing activities along with bolus volume and material consistency were also the subject of another study using a microphone and 2 sEMG channels [24]. The use of sEMG with microphone improved the accuracy from 73 to 84%.

This study introduces a non-invasive swallowing sensing technique that relies on a single-channel sEMG and a wrist-worn IMU sensor to monitor and evaluate the ingestive activities. This study also provides an sEMG-based algorithm to quantify food bolus through using three different food classes and drink sip volumes.

## Results

A total of 4675 swallows (2200 water swallows and 2475 solid material swallows) were collected. 335 swallows (200 water swallows and 135 solid material swallows) were then excluded due to low signal quality and noise dominance that occurred during the data collection procedure.

**Fig. 1:**
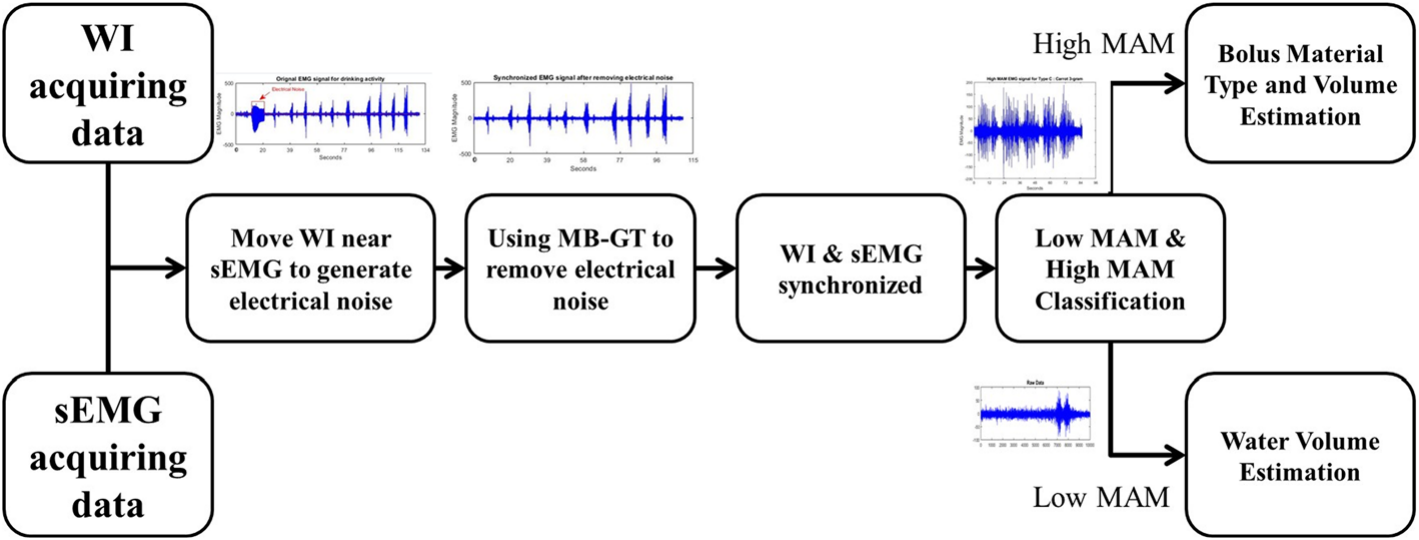
A block diagram that shows overview of pipeline for the proposed method. This pipeline includes the process of end-to-end synchronization and fusion between the data from the WI and sEMG sensors

**Fig. 2:**
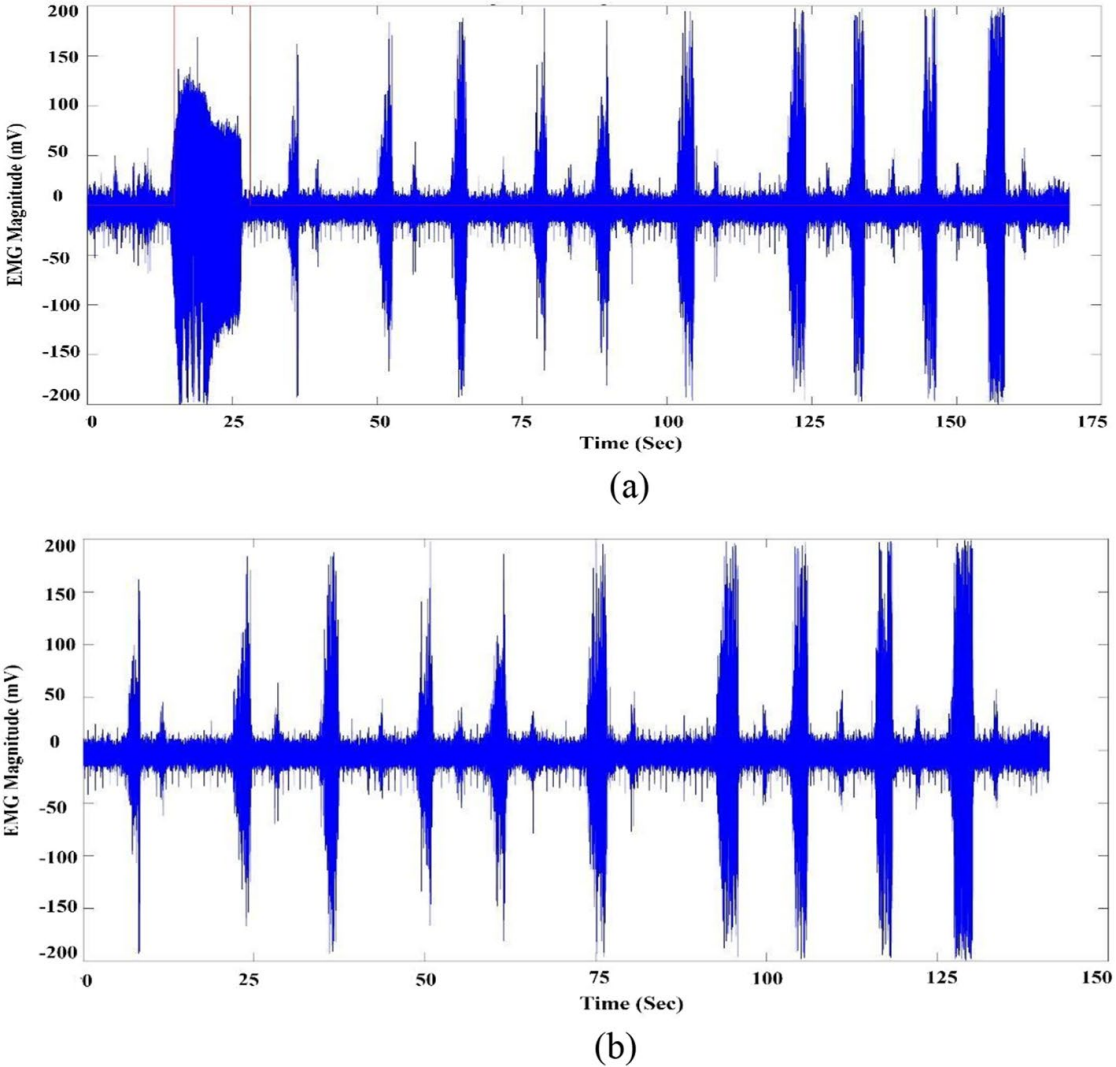
**a** sEMG with electrical high-frequency interference “red line represents the MB-GT detection window”. **b** sEMG signal after removing electrical interference

Swallowing signals were collected via two wearable sensors, a mobile BITalino single electrode sEMG and a wrist-mounted IMU (WI) sensor. To guarantee the synchronization of collected signals, the WI and sEMG sensors were started at approximately the same time. Furthermore, the participants were also asked to move the WI sensor close to the sEMG to induce some electrical interference that appears as high-frequency (sudden) change in the sEMG signal. This sudden change was detected and used to perform end-to-end synchronization between the signals from the two sensors prior to analysis by using Memory Based Graph Theoretic technique (MB-GT). This technique is considered a fast adaptive algorithm for abrupt change detection [25]. After detecting and removing the noise window, both WI and sEMG signals were considered in alignment. The full processing chain is shown in Fig. 1. The sEMG signals before and after synchronization are shown in Fig. 2.

### Solids vs water swallows prediction

**Fig. 3:**
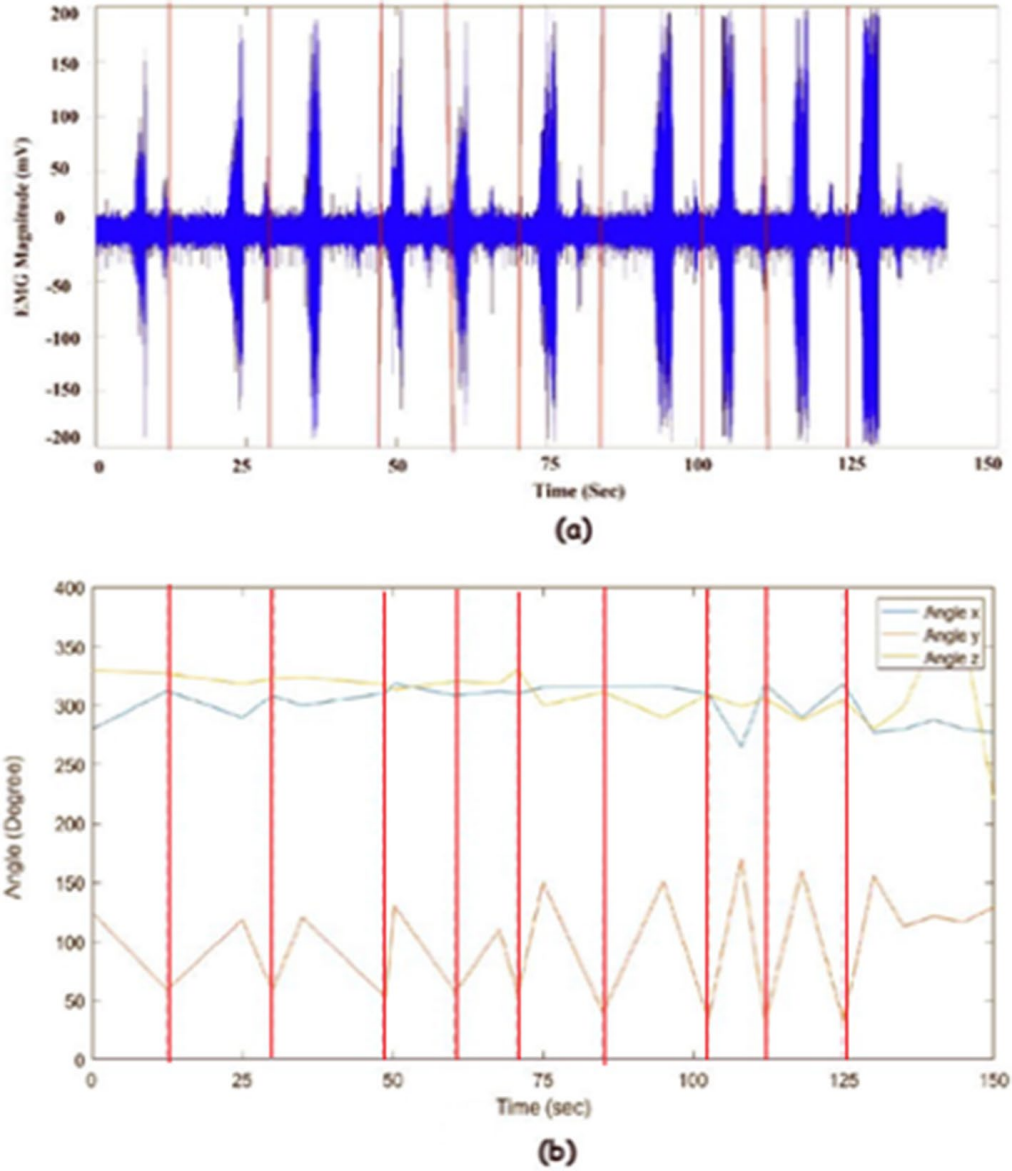
**a** sEMG and **b **WI signals fusion to indicate the ingestive activities “the red lines represent the start of ingestive intake”

After signals from WI and sEMG were aligned temporally as described previously, these signals were fused to identify and remove no-activity periods (periods of “no ingestive intake”) as only periods of hand motion in WI signals indicate solid/water intake. We described the solid or liquid intake using the IMU’s X, Y, and Z rotation angles. Prior to the experiments, we verified the subjects’ angles for both eating and drinking. Then, we employed thresholding and decision trees to ascertain the angles for each. There are specific X, Y, and Z rotation angles for every subject in every activity. The majority of subjects use the same angles for each eating or drinking activities, with a difference between the X and Y angles in both activities. The X and Y angles for drinking were located between 300 and 350 and 1 and 60, respectively. Additionally, the X and Y angles for eating were located between 200 and 300 and 90 and 150, respectively. WI was able to use rotation angles to differentiate between solid and water input based on the prior angles. Following temporal alignment with sEMG, the activity times marked in the WI signals were converted to sEMG using the time stamp in WI and frequency in sEMG. An example of signal-fusion outcome is shown in Fig. 3. The red lines represent the onset of ingestive activities. Signal properties of sEMG differ in magnitude and duration according to the underlying activity performed by the subject.

The muscle activity magnitude (MAM) can be divided into either high MAM or low MAM [26]. Low MAM segments of the sEMG represent swallow events only, which correspond to fluid and saliva swallowing. On the other hand, high MAM segments include biting, chewing, and swallowing events, which correspond to eating activities. The segments of sip–swallow and bite–chew–swallow events were further identified through applying the MB-GT algorithm on sEMG in the duration between each two successive eating/drinking events, as identified by WI. As mentioned previously, MB-GT is an efficient technique for detecting abrupt changes in biosignals [25, 27].

### Bolus material type and volume estimation

**Fig. 4:**
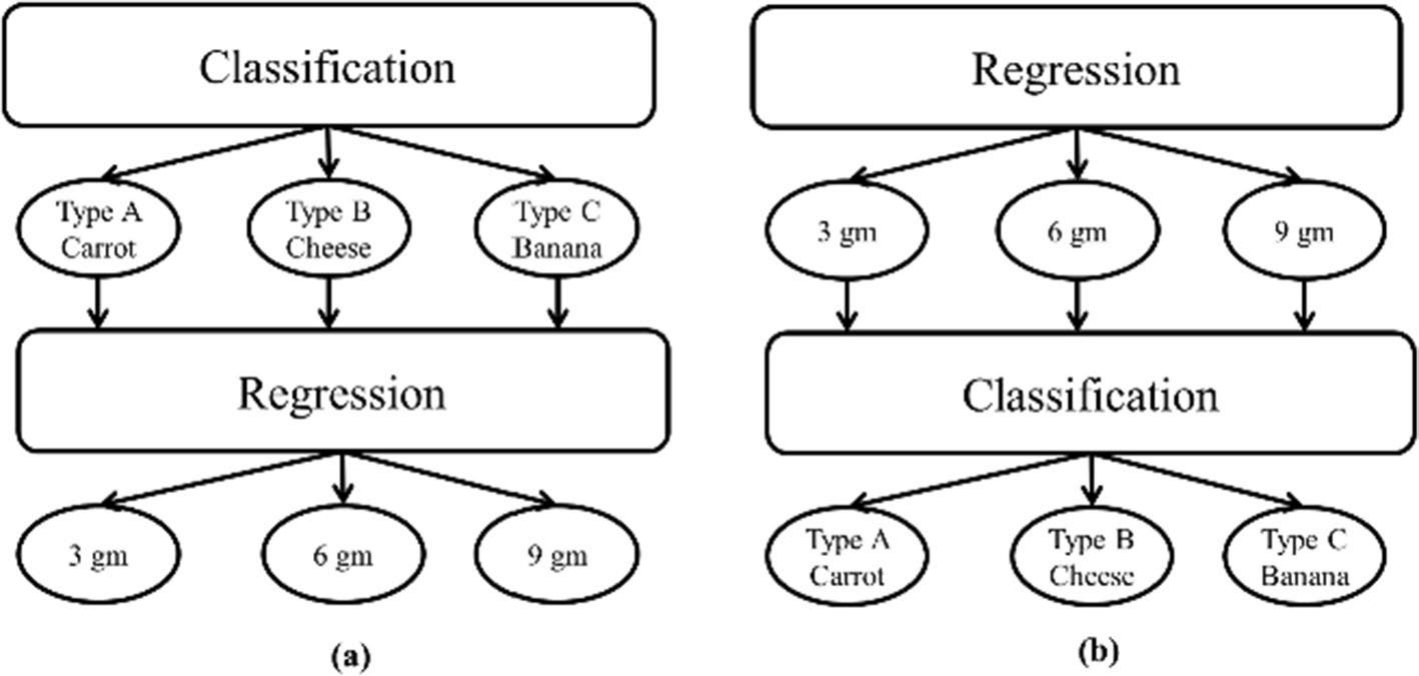
A block diagram that shows the two approaches for bolus material type and volume estimation. **a** First Approach, **b** Second Approach.

In high MAM segments, to determine the solid material type and volume we divided the problem into two main steps: (1) classification for identifying the material type and (2) regression for volume quantification. We used two different approaches to implement both steps: starting with classification then doing regression and vice versa, as illustrated in Fig. 4.

For the classification task that determines the solid material type, our experiment included 3 classes representing different softness levels: Type A, carrot (hard texture); Type B, cheese (intermediate texture); and Type C, banana (soft texture).

**Fig. 5:**
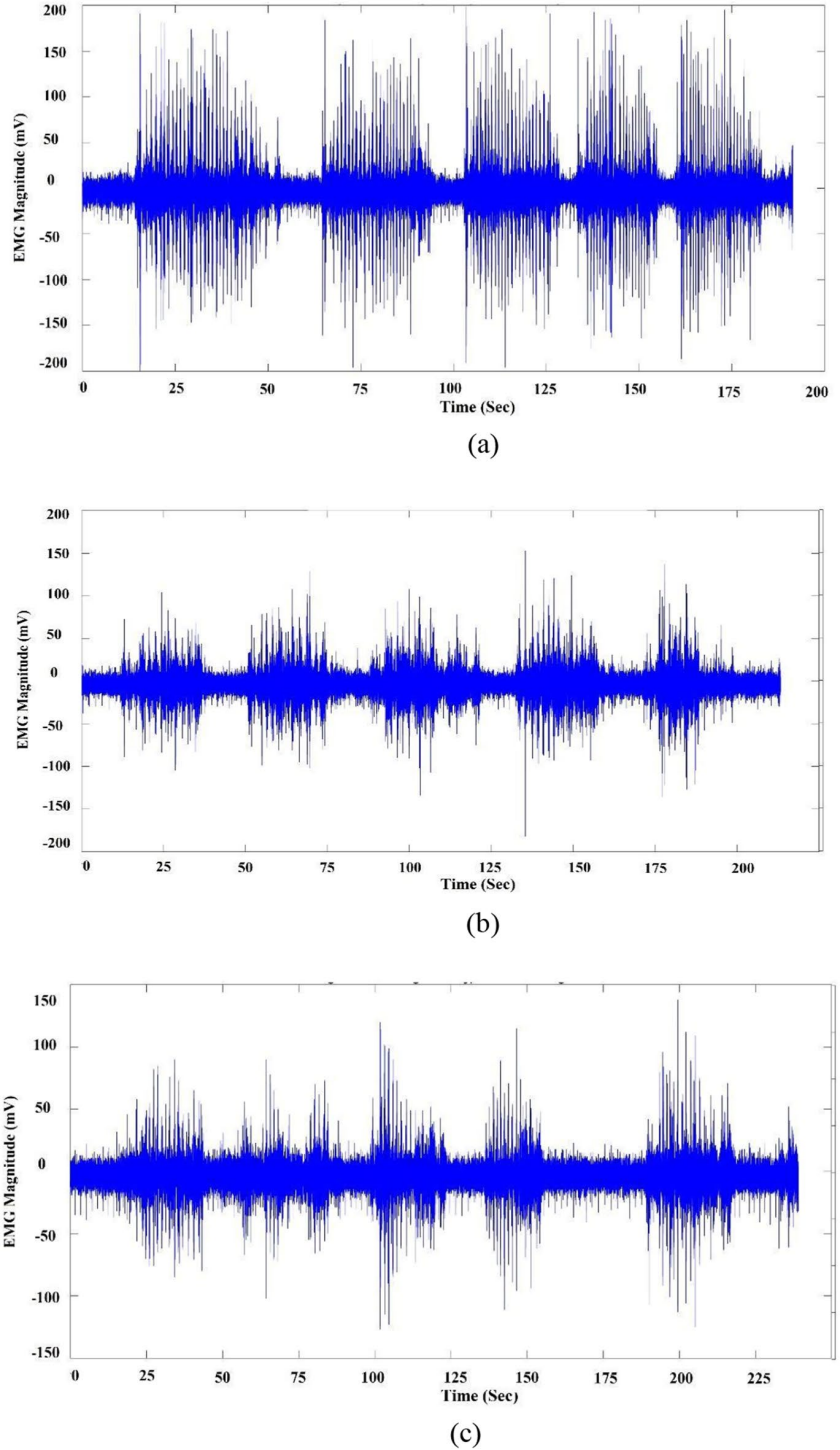
EMG signals for the different classes: **a** carrot, **b** cheese, and **c** banana

**Fig. 6:**
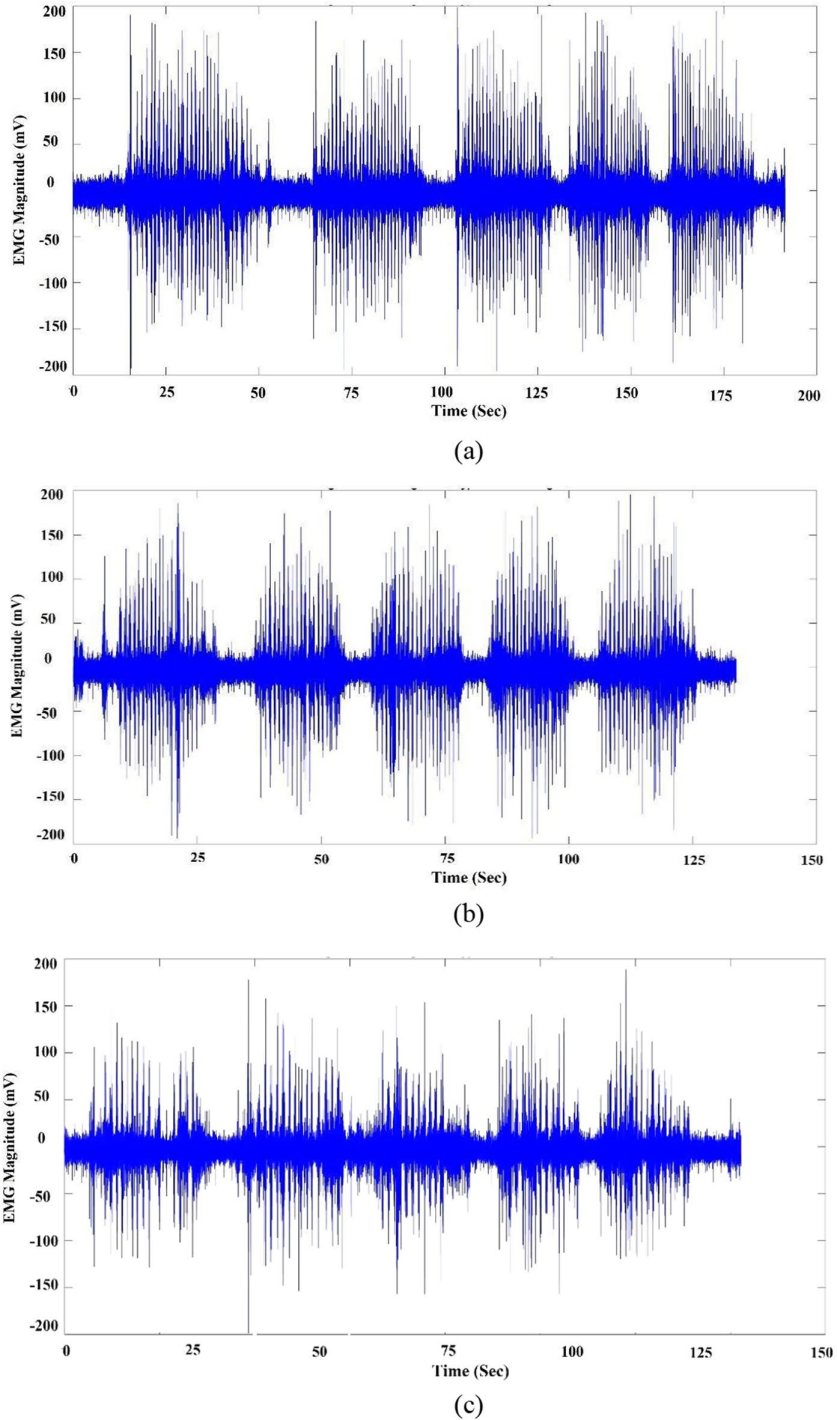
EMG signals for different carrot bolus sizes: **a** 9 gm, **b** 6 gm, and **c** 3 gm

Examples of sEMG signals for the three classes are shown in Fig. 5, while examples for signals from different bolus weights for “carrots” are shown in Fig. 6.

Forty features calculated in time–frequency domains commonly used with EMG signals in the literature, were chosen for our study [28–30] and are shown in Table 1.

**Table 1:** EMG selected features

No.	Feature	No.	Feature
1	Enhanced Mean absolute value	21	Skewness
2	Enhanced Wavelength	22	Kurtosis
3	New Zero Crossing	23	Coefficient of Variation
4	Absolute Value of Summation of exp root	24	Standard Deviation
5	Absolute Value of Sum of Square Root	25	Variance
6	Mean Value of Square Root	26	Average Energy
7	Log Teager Kaiser Energy Operator	27	Integrated EMG
8	Log Coefficient of Variation Cardinality	28	Mean Absolute Value
9	Log Difference Absolute Standard Deviation Log	29	Slope Sign Change
10	Difference Abs. Mean Value	30	Zero Crossing
11	Difference Variance Value	31	Waveform Length
12	V-Order	32	Root Mean Square
13	Temporal Moment	33	Average Amplitude Change
14	Difference Absolute Mean Value	34	Difference Absolute Standard Deviation
15	Auto-Regressive Model	35	Value
16	Mean Absolute Deviation	36	Log Detector
17	Interquartile Range	37	Modified Mean Absolute Value
18	Variance of EMG	38	Modified Mean Absolute Value 2
19	Willison Amplitude	39	Myopulse Percentage Rate
20	Maximum Fractal Length	40	Simple Square Integral

The bites data were split into two groups (80–20%): 1872 bites were used for training and 468 bites were used for testing. Since sEMG data usually suffer from inter-subject variability, we used two validation methods.

First, both training and testing data were sampled evenly from all subjects (among-subjects validation or AS) and, second, training and testing data were sampled from different subjects (cross-subject validation or CS). The data splitting process was repeated 5 times until all data points were included at least once in the training and testing process (fivefold cross-validation). *K*-Nearest Neighbor (KNN) classifier, support vector machine (SVM) and DT were tested as classifiers to determine the bolus class. KNN was configured to use *K* = 5 and Euclidian distance; while SVM was configured to use *C* = 1, a Radial Basis Function (RBF) kernel. For DT, entropy was employed as a metric to assess the efficacy of each split, with a stipulation that an internal node must have at least 2 samples to be eligible for splitting. Additionally, the expansion of nodes continued until all leaves became pure.

For the regression task that determines the bolus volume, the bites included three different weights: 3, 6, and 9 g for each type. The material type was known at this stage given that this regression task is performed after the classification task. DT, extra trees (ET), AdaBoost (AB), gradient boosting (GB), eXtreme gradient boosting (XGB), light gradient boosting (LGB), SVM, and Gaussian regressors (GPR) were used and validated in a fivefold cross-validation manner. Further, a two-layer feed-forward neural network was tested with both Levenberg–Marquardt (LM) and Bayesian regularization (BR). The neural network consisted of a hidden layer containing 50 neurons, an output layer with a single linear rectified neuron to represent the regression output, and it underwent 1000 epochs during training.

In this approach, KNN and DT showed the highest accuracies in the AS validation scheme, while SVM showed better performance in the CS validation scheme as shown in Table 2, with sensitivity and specificity values of 92.3 and 96.1%, respectively.

**Table 2:** Solid material type classification accuracies using different validation schemes

Validation scheme	KNN (%)	SVM (%)	DT (%)
AS	100	99.5	100
CS	89.32	94.4	86.1

**Fig. 7:**
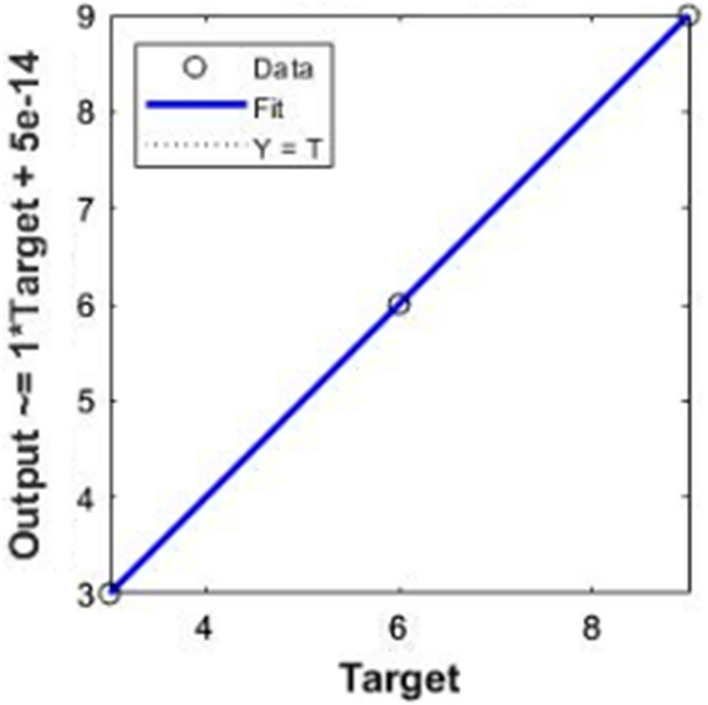
ET regression model for Class A

Table 3 depicts the root mean squared error (RMSE) for the regression task that determines the bolus volume. While ET showed the best overall performance for both AS and CS validation in the three material types, most of the tested regression models showed close performances. Table 4 shows the detailed performance measures for ET regression results including the mean absolute error and R^2^ for each material type. Also Fig. 7 shows the ET regression fitting model.

**Table 3:** RMSE for bolus volume estimation results in different validation schemes for each class

Material type		DT	SVM	GBR	NN-LM	NN-BR	ET	AB	RF	GB	XGB	LGB
A	AS	0.72	0.48	0.25	0.65	0.025	0	1.28595	0.0684	0.35752	0.00037	0.05078-
	CS	2.22	2.54	1.97	3.52	2.85	4.83056	4.83293	3.66575	4.7686	4.6351	4.81869
B	AS	0.45	0.51	0.05	0.45	0.1	0	− 0.85676	− 0.00832	0.09599	0	− 0.01746
	CS	2.54	2.8	2.72	3.5	2.28	2.95479	2.96138	3.17588	3.02591	2.91991	3.09822
C	AS	0.52	0.66	0.34	0.66	0.9	0	1.28595	0.0684	0.35752	0	0.03674
	CS	1.88	1.72	1.4	4.6	1.45	0.56471	1.40598	0.58727	0.68823	0.56473	0.57472

**Table 4:** Bolus volume estimation results for the lowest error regression schemes (ET) of each class

Material type	Validation scheme	RMSE	MAE	*R*2
A	AS	0	0	1
	CS	4.83056	4.17036	0.59288
B	AS	0	0	1
	CS	2.95479	2.62233	0.46163
C	AS	0	0	1
	CS	0.56471	0.56473	0.94685

As a second approach, we performed the regression first followed by classification to test the effect of the pre-knowledge of bolus volume/weight on the classification results of the material type. For this purpose, we grouped the results of regression task into 3 groups small volume (< 4.5), intermediate volume (> 4.5 and < 7.5) and large volume (> 7.5). Then followed by bolus type classification for each regression group. Tables 5 and 6 show the results and the top performing models in the regression and classification tasks of the experiments performed in the second approach. We can clearly see the deterioration in the regression error and classification accuracy for this approach compared to the first approach which indicates that the pre-knowledge of the material type is a substantial factor that enhances the quality of bolus volume estimation. Other regression models tested in this approach showed poorer results, therefore they were removed from Table 5.

**Table 5:** RMSE for bolus volume estimation in different validation schemes for the second approach

Validation scheme	DT	SVM	GBR	NN–LM	NN–BR
AS	2.3	3.01	2.33	4.7	3.6
CS	4.5	4.2	3.01	6.3	6.32

**Table 6:** Bolus material type classification accuracies in different validation schemes for each class in the second approach

Bolus size	Validation scheme	KNN (%)	SVM (%)	DT (%)
Low volume	AS	84	86.3	84.2
	CS	80	73.9	71.2
Intermediate volume	AS	71	72.2	72.8
	CS	69.8	79	70.4
High volume	AS	93	95	96.5
	CS	86	86.6	92.2

### Water sip volume estimation

**Fig. 8:**
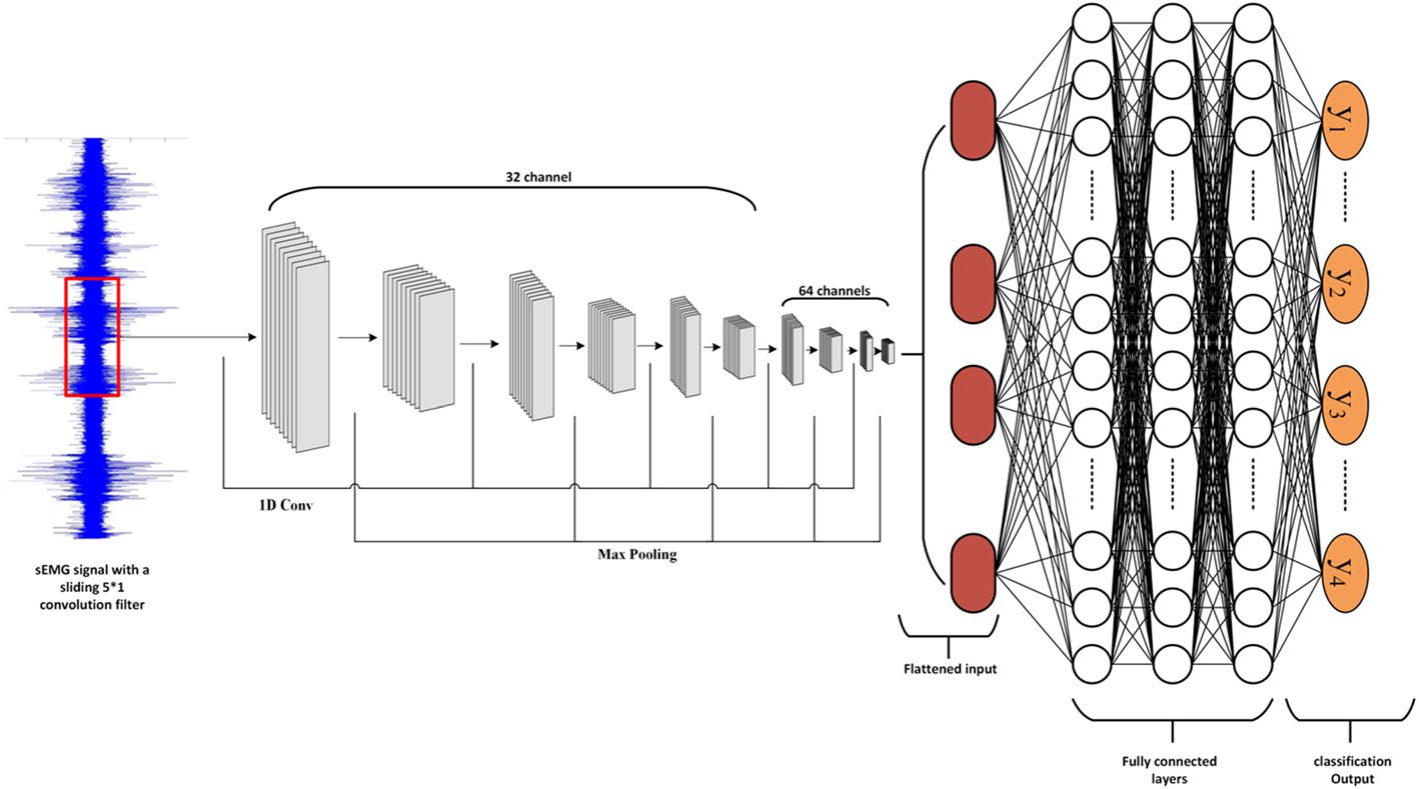
Architecture of the convolutional neural network used for water sip volume classification. 5 1D convolutional layers each followed by batch normalization and maxpooling (1*2 on time dimension). The output of the last convolutional layer is then flattened and fed into two dense layers and an output layer to generate the classification result

In this part of the study, the goal was to estimate the water sip volumes using low MAM signals via two methods. The first method employed regression approach for a direct estimate of the sip volume using the 40 features described before and the second method utilized a deep convolutional network that used raw sEMG signals to classify sip volume into one of four classes (10, 20, 30, and 40 ml). The deep network (Fig. 8) consisted of 5 1D convolutional layers, each with a kernel size of 5 and a number of output channels of 32, 32, 32, 64, and 64, respectively. Each convolutional layer was followed by batch normalization and 1D maxpooling (of size 1*2). The convolutional network was followed by two dense layers to combine the features and generate the classification output.

Table 7 shows the RMSE values for each of the tested regression models in the first approach task for AS and CS validation. We can see that neural networks-based regression with Bayesian regularization gives the best performance among the other regression models, with RMSE values of 0.57 and 4.1 for AS and CS validation scheme, respectively.

**Table 7:** RMSE for water volume estimation using different regression schemes

Validation scheme	DT	SVM	GBR	NN–LM	NN–BR	ET	AB	RF	GB	XGB	LGB
AS	3.2	2.5	1.7	0.29	0.57	0.0	7.99	0.897	4.47	0.028	0.78
CS	6.2	7.2	5.8	6.4	4.1	14.57	12.61	14.49	13.77	14.57	14.55

In the second approach, the deep network that used raw sEMG signals showed an accuracy of up to 99% while validating AS and 66% while validating CS.

## Discussion

We presented a system and processing methods for the detection and quantification of swallowing activities of an individual in a manner that does not require manual logging. The approach used in this study employed a less-controlled environment compared to previous studies [31]. It also provided methods for quantification of ingested solid and fluid materials. These combined abilities bring this research one step closer to achieving an environment where many aspects of the ingestive behavior of an individual can be characterized in the least possible intrusive way. Such a goal can prove invaluable for many clinical and day-to-day living cases, enabling better care for the elderly, diabetics, and those seeking better weight control in a self-guided manner or via the help of medical professionals, to list a few.

To carry out this work, we relied on a single-channel surface electromyography sensor affixed on the sternocleidomastoid muscle in the neck area. We tested various machine learning techniques for binary classification of swallowing vs. non-swallowing and solid vs. fluid intake. The use of sEMG proved valuable in terms of minimum interference and reliability, despite lack of ideal signal-to-noise ratio conditions due to the small size of the target muscle and the limited capabilities of the used device. Nevertheless, the accuracy of swallowing vs. non-swallowing classification was above 99% and the accuracy of solid vs. fluid classification was 96% as shown in this and previous studies [31].

The two main goals of this research, however, were the quantification of solid bolus type and weight and the quantification of the liquid intake volume as one of four pre-selected values. The selection of three solid types was rather intuitive to cover different “softness” levels: soft, medium, and hard. The performance of the solid type classification was above 99.5%, while root mean squared error of quantity estimation of each type from the possible three weight values was 0.00037. This demonstrates that it is possible to use sEMG to estimate the type of ingested solid material and quantify its weight with high accuracy and low RMSE. The achieved accuracies of using classification then regression were low compared to regression then classification as shown in Tables 2 and 6.

For liquid volume estimation, we pre-selected four different volume sizes that lend themselves well to model the lowest and highest sip volume that the subject could swallow at once. The accuracy of this part ranged between 100 and 66% for among and cross-subject validation schemes. The drop in classification/regression performance in the different presented tasks can be accounted for by the widely known high inter-subject variability in sEMG. Therefore, a future direction to boost the cross-subject performance of this platform could be considering different pre-processing strategies of the sEMG signals and using a variety of features to reduce the variability.

The use of the wrist-mounted IMU sensor was another tool that helped reduce the complexity of the problem in terms of demarcation of onset of about-to-eat or about-to-drink moments as a facilitator for data segmentation. The performance of this type of sensors is expected to be moderate to good even in less-controlled environments.

The main limitations of this study are: the limited types and quantities of solids and liquids, short experiment duration and limited number of visits. Hence, future work should include: more types and quantities of solids and liquids, and moving to free-living environments.

## Conclusions

The goal of this work was to detect and measure an individual's swallowing behaviors in a way that did not require human logging. Through the wrist angles, the WI was able to successfully identify ingestive intake, whether it was eating or drinking. With an R2 value approaching one and root mean squared error values as low as 0.00037, the system was able to quantify food intake. In the case of fluid consumption, multiple approaches, including regression and classification models, were used to estimate bolus volumes. Among the investigated models, neural networks-based regression had the highest R2 and the lowest root mean squared error (0.2). The employment of a simple EMG-based system and wrist-mounted IMU to monitor ingestive behavior with a simple classification algorithm that can be quickly constructed utilizing modest computing platforms makes practical implementation of this methodology in real life highly feasible.

## Methods

### Experiment protocol and signal acquisition

This study was approved by the institutional review board of Cairo University and all procedures were performed in accordance with the 1964 Helsinki Declaration and the Nuremberg code of ethics and their later amendments or comparable ethical standards [32]. All participants provided informed written consents prior to enrollment in the study including consent to publish. Fifty-five healthy individuals (25 males, 30 females, age: 23.7 ± 5.7 years, BMI: 26.7 ± 10.83 kg/m^2^) participated in the experiment.

Participants were deemed eligible based on the following inclusion criteria: no history of medical conditions that affect swallowing and/or food intake. Each participant performed the experiment in a one one-hour session where three activities were investigated: drinking, eating, and talking. Drinking activities included 40 water sips equally split among 4 fixed volumes (10, 20, 30, and 40 ml), for a total volume of one liter per participant. Eating activities included administering 45 boluses equally split among 3 materials: banana, cheese and carrot and 3 weights per each type: 3, 6, and 9 gm. Bolus volume and weight were controlled and verified through using graded cups and a digital kitchen scale prior to starting each experiment session. A total of 4675 swallows (2200 water swallows and 2475 solid material swallows) were collected. 335 swallows (200 water swallows and 135 solid material swallows) were then excluded due to low signal quality and noise dominance that occurred during the data collection procedure.

**Fig. 9:**
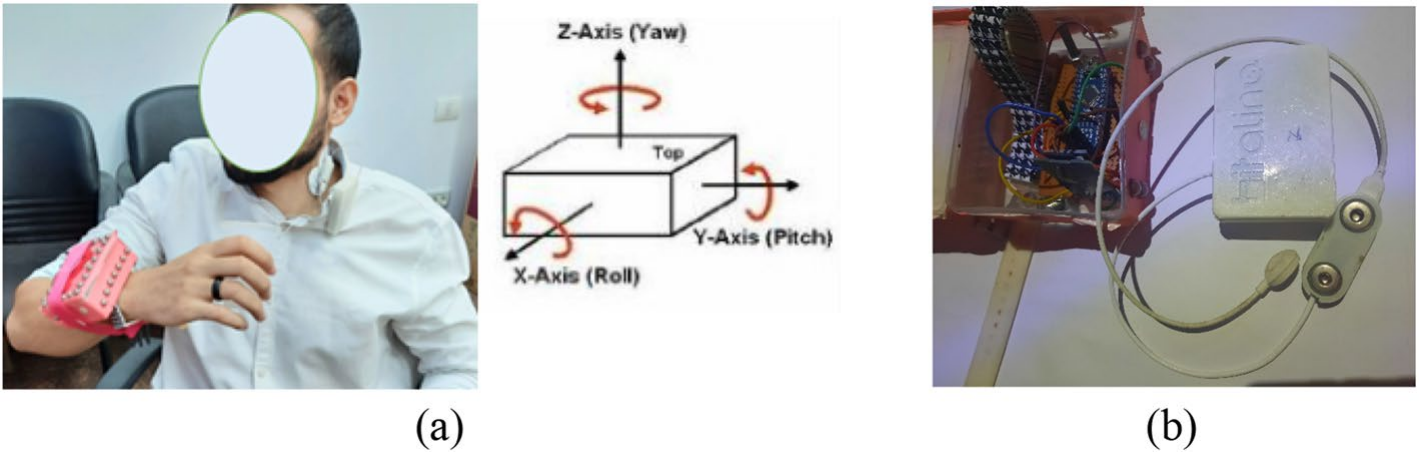
**a** Subject wearing system. **b** Sensors utilized in the study including the WI in a case (the red box on the left) which is attached to the wrist of the subject and sEMG BITalino (on the right)

Swallowing signals were collected via two wearable sensors, a mobile BITalino single electrode sEMG and a wrist-mounted IMU (WI) sensor. The BITalino revolution kit is a Bluetooth compact biosignals platform designed for research purposes (range: up to ~10 m (in line of sight)) [33]. Sampling rate of BITalino was 1000 Hz with Battery 500 mA, 3.7 V, LiPo (rechargeable). The sEMG electrode (Fig. 9) was placed on the left sternocleidomastoid muscle and the signals were acquired using the BITalino open-source software. The left sternocleidomastoid muscle was chosen as it is considered least uncomfortable neck location to place the electrodes during swallowing plus it is proven in previous studies to produce swallowing signals of better quality. An MPU-6050 sensor was used as the IMU module on the wrist (Fig. 9) [34]. The IMU module was used to detect the angle of tilt or inclination along the X, Y, and Z axes of the wrist shown in Fig. 9a. Wrist angles data from the IMU sensor were transferred to an Arduino Nano kit through an I2C interface and then written into an SD card by the Arduino kit. The 3 axes of activities from the WI were sampled at 1 kHz and the periods of ingestive intake were identified through hard-thresholding and decision trees (as shown Fig. 3). At the beginning of each session, and as a calibration step, each subject was asked to eat and drink freely to determine the range of the wrist angles for both eating and drinking activities. A threshold was determined for each axis and a DT was later used to demarcate the periods of activity and the type of activity (eating or drinking) [27].

## Data Availability

The datasets used and/or analyzed during the current study are available from the corresponding author upon reasonable request.

## Abbreviations

sEMG: Surface electromyogram
WI: A wrist-mounted IMU
MB-GT: Memory Based Graph Theoretic technique
DT: Decision tree
ET: Extra trees
AB: AdaBoost
GB: Gradient boosting
XGB: EXtreme gradient boosting
LGB: Light gradient boosting
SVM: Support vector machine
GBR: Gaussian regressors
NN-LM: Neural network with Levenberg–Marquardt
NN-BR: Neural network with Bayesian regularization
RMSE: Root mean square error
R^2^: Coefficient of determination
